# SARS Unique Domain (SUD) of Severe Acute Respiratory Syndrome Coronavirus Induces NLRP3 Inflammasome-Dependent CXCL10-Mediated Pulmonary Inflammation

**DOI:** 10.3390/ijms21093179

**Published:** 2020-04-30

**Authors:** Young-Sheng Chang, Bo-Han Ko, Jyh-Cherng Ju, Hsin-Hou Chang, Su-Hua Huang, Cheng-Wen Lin

**Affiliations:** 1 Department of Medical Laboratory Science and Biotechnology, China Medical University, Taichung 404394, Taiwan; a0989016192@gmail.com (Y.-S.C.); pig60666@gmail.com (B.-H.K.); 2 Graduate Institute of Biomedical Sciences, China Medical University, Taichung 404394, Taiwan; jcju@mail.cmu.edu.tw; 3 Department of Molecular Biology and Human Genetics, Tzu Chi University, Hualien 970301, Taiwan; hhchang@mail.tcu.edu.tw; 4 Department of Biotechnology, Asia University, Wufeng, Taichung 413305, Taiwan; shhuang@asia.edu.tw

**Keywords:** SARS-coronavirus, SARS-CoV unique domain (SUD), CXCL10, NLRP3 inflammasome, pulmonary inflammation

## Abstract

Severe acute respiratory syndrome–associated coronavirus (SARS-CoV) initiates the cytokine/chemokine storm-mediated lung injury. The SARS-CoV unique domain (SUD) with three macrodomains (N, M, and C), showing the G-quadruplex binding activity, was examined the possible role in SARS pathogenesis in this study. The chemokine profile analysis indicated that SARS-CoV SUD significantly up-regulated the expression of CXCL10, CCL5 and interleukin (IL)-1β in human lung epithelial cells and in the lung tissues of the mice intratracheally instilled with the recombinant plasmids. Among the SUD subdomains, SUD-MC substantially activated AP-1-mediated CXCL10 expression in vitro. In the wild type mice, SARS-CoV SUD-MC triggered the pulmonary infiltration of macrophages and monocytes, inducing CXCL10-mediated inflammatory responses and severe diffuse alveolar damage symptoms. Moreover, SUD-MC actuated NOD-, LRR- and pyrin domain-containing protein 3 (NLRP3) inflammasome-dependent pulmonary inflammation, as confirmed by the NLRP3 inflammasome inhibitor and the NLRP3^−/−^ mouse model. This study demonstrated that SARS-CoV SUD modulated NLRP3 inflammasome-dependent CXCL10-mediated pulmonary inflammation, providing the potential therapeutic targets for developing the antiviral agents.

## 1. Introduction

Severe acute respiratory syndrome (SARS)-associated coronavirus (SARS-CoV) is a highly contagious agent that causes severe lung damage, including bronchial epithelial dysfunction, pulmonary infiltration with immune cells, and even lung fibrosis [1,2,3,4]. Besides SARS-CoV, there are five well known human coronaviruses (HCoVs), such as HCoV-OC43, HCoV-NL63, HCoV-HKU1, Middle East respiratory syndrome CoV (MERS-CoV), and coronavirus COVID-19 (formerly known as Wuhan coronavirus and 2019-nCoV) [5]. Among them, SARS-CoV, MERS-CoV, and COVID-19 are highly pathogenic HCoVs, and their spread initiated global outbreaks with high fatality rates in 2003, 2012, and 2020, respectively [6,7]. SARS-CoV infected 8096 confirmed cases with 774 deaths in the outbreak of 2002-2003 [8]. MERS-CoV has caused a global spread since June 2012, with 2494 infected patients including 858 deaths until February 2020 (https://www.who.int/emergencies/mers-cov/en/). Nowadays, COVID-19 has caused a big outbreak in China and is spreading globally by a limited people-to-people transmission, resulting in 2,319,066 confirmed cases including 157,920 deaths in 213 countries, areas or territories, with cases during the period from 31st December 2019 to 21st April 2020 (https://www.who.int/emergencies/diseases/novel-coronavirus-2019). The mortality rate of these pathogenic coronaviruses ranges from 6.8% for COVID-19 patients to 35% for MERS patients.

SARS-CoV prompts a cytokine storm in the lung tissue and systemic circulation, linking with the lung injury in SARS patients, such an immune cell infiltration at the early phase and pulmonary fibrosis at the late phase [9,10]. Gene signatures in the lung of SARS patients indicate that CXCL10, CCL2, interferon-α/β receptor 1 (IFNAR1), Interferon gamma receptor 1 (IFNGR1), and cluster of differentiation 58 (CD58) mRNAs are persistently generated during the infection [11]. Cytokine profiles in the plasma of SARS patients reveals that the proinflammatory CC and CXC chemokines CCL2, CCL3, CCL5, and CXCL10 level in infected patients is higher than in healthy controls. Meanwhile, the infected patients who died exhibit significantly higher levels of CXCL10 than those patients who recovered [9]. Moreover, an increase in the plasma level of CXCL10 is found in the severe pneumonia patients infected with MERS-CoV and COVID-19, which is linked with the infiltration of immune cells into alveolar space, peribranchial, and perivascular of the lung [12,13]. Therefore, the dysregulation of CXCL10 expression in the lung tissue and systemic circulation during infection could play an essential role in the pathogenesis of pathogenic coronaviruses. 

HCoVs contain a single-stranded and positive-sense RNA genome of near 30 kb with a 5′ cap and a 3′ poly–(A) tract, encoding two replicase polyproteins pp1a and pp1ab, spike (S), envelope (E), membrane (M), nucleocapsid (N) and several accessory proteins [1,2,14,15]. The replicase polyproteins pp1a and pp1ab are cleaved by two viral proteases papain-like (PLpro) and 3C-like (3CLpro) to yield 16 non-structural proteins (Nsp1–Nsp16), which are responsible for viral genome replication. SARS-CoV Nsp3 is the largest Nsp comprising several domains, e.g., An N-terminal Glu-rich acidic domain (also called Nsp3a), an X domain with poly(ADP-ribose) binding properties (Nsp3b), the SARS-CoV unique domain (SUD) (Nsp3c), papain-like protease (PL2^pro^, Nsp3d), non-canonical papain-like domain, and a Y domain (Nsp3e) [16]. SARS-CoV unique domain (SUD) appears in SARS-CoV, as also found in other coronaviruses like MERS CoV and COVID-19. Moreover, amino acid alignment of SARS-SUD with MERS-CoV and COVID-19 shows 15% and 75% identity, respectively. SUD contains three macrodomains (SUD-N, SUD-M, and SUD-C) with the structure similarity to the X-domain (N3), exhibiting a specific affinity for oligo(G) nucleotides (both deoxynucleotides and ribonucleotides) [17]. The SUD-M domain is a fundamental component of the replication/transcription complex, involving in SARS-CoV genome replication and transcription [18]. SUD also acts as an enhancer to reinforce the interaction between SARS-CoV Nsp3 and E3 ubiquitin ligase ring-finger and CHY zinc-finger domain-containing 1 (RCHY1), and precedes RCHY1-mediated ubiquitination and degradation of p53 [19]. In addition, SUD binds with the G-stretches within the 3′-nontranslated regions of mRNAs of host cell genes like Bbc3, RAB6B, and TAB3 [20]. TAB3, a partner of TAK1 kinase, activates AP-1- and NF-κB-dependent genes, such as CXCL9, CXCL10, and CCL8 [21]. Therefore, further examining the possible involvement of SUD in CXCL10-mediated SARS pathogenesis is needed. 

This study investigates the functional activity and mechanism of SUD in CXCL10-mediated pulmonary inflammation using in vitro and in vivo assays. To evaluate the mechanism of CXCL10 up-regulation, human lung epithelial cells with transiently transfection and stably expression of SARS-CoV SUD were examined using real-time PCR plus specific inhibitors, dual luciferase reporter system, and nuclear translocation of transcriptional factors. The mouse model via the intratracheal instillation with recombinant plasmids was established for elucidating SUD-induced CXCL10-mediated pulmonary inflammation.

## 2. Results 

### 2.1. SUD-MC Subdomain Up-Regulated the Expression of CXCL10 in Human Lung Epithelial Cells

**Figure 1:**
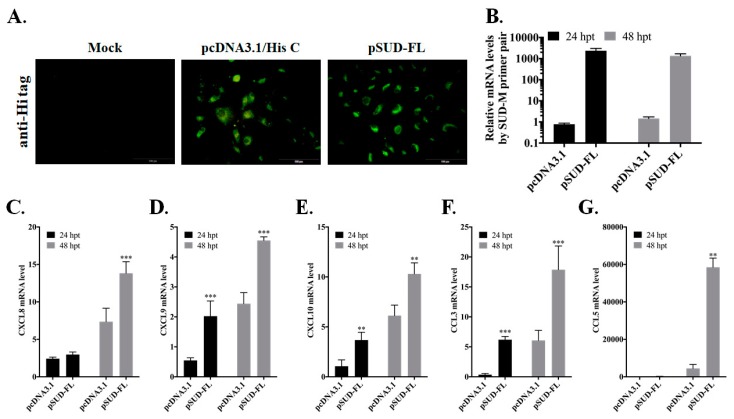
Chemokine profile of full-length SUD-expressing alveolar epithelial cells. A549 cells were transiently transfected with pcDNA3.1/His C or pSUD-FL, and harvested 24 h and 48 h post transfection. The transfected cells were performed using immunofluorescent staining with anti-His mAb and secondary antibodies conjugated with FITC (**A**). Total RNAs of transfected cells were isolated and analyzed by quantitative real-time PCR. Relative mRNA levels of SUD-M (**B**), CXCL8 (**C**), CXCL9 (**D**), CXCL10 (**E**), CCL3 (**F**), and CCL5 (**G**) were normalized by β-actin mRNA, presented as relative ratio. **, *p* value < 0.01; ***, *p* value < 0.001 compared with pcDNA3.1/His C transfected cell. Scale bar, 200 μm.

To clarify the role of SUD in pulmonary inflammation during SARS-CoV infection, transient transfection of recombinant pSUD-FL plasmid in human alveolar basal epithelial A549 cells was performed to examine the expression profile of chemokines (Figure 1). Real-time PCR assay indicated that transiently transfected cells with pSUD-FL, expressing the proteins (Figure 1A) and mRNAs (Figure 1B) of full-length SUD, had a significant up-regulation of CXCL9, CXCL10 and CCL3 mRNAs in comparison to empty-vector transfected cells 24 and 48 h post transfection (Figure 1D–F). The results displayed that SARS-CoV SUD was one of the key viral proteins for initiating the production of inflammatory chemokines in lung epithelial cells.

**Figure 2:**
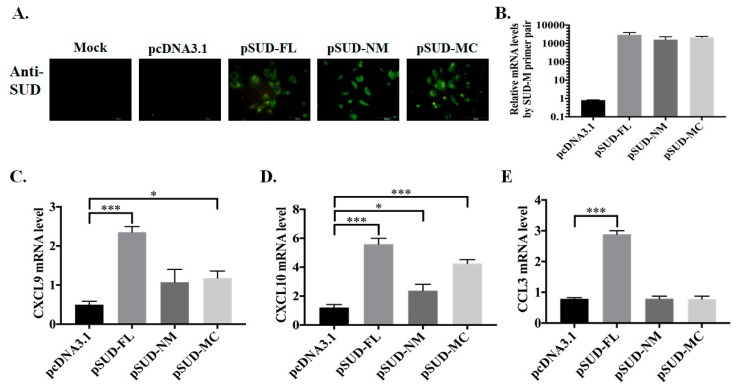
Chemokine profiles of human lung epithelial cells in responses to SUD subdomains. Stably transfected A549 cell lines expressing full-length SUD, SUD-NM and SUD-MC, respectively, were used to examine the protein and mRNA expression of indicated SUD subdomains using immunofluorescent staining (**A**) and real-time PCR (**B**). Moreover, relative mRNA levels of CXCL9 (**C**), CXCL10 (**D**), and CCL3 (**E**) in the stable cell lines were determined after normalized by β-actin mRNA. *, *p* value < 0.05; ***, *p* value < 0.001 compared with the stable cell line transfected with pcDNA3.1/His C. Scale bar, 50 or 100 μm.

To examine the activity of NM and MC subdomains of SARS-CoV SUD in activating the chemokine expression, the stable clones transfected with pcDNA3.1/His C, pSUD-FL, pSUD-NM, and pSUD-MC, respectively, were generated after three weeks of G418 selection. Immunofluorescence staining with SUD-immunized sera and quantitative RT-PCR assay indicated the protein and mRNA expression of SUD and its subdomains in indicated stable clones (Figure 2A,B). Chemokine expression profile revealed that SUD-MC expression caused a higher increase of CXCL10 mRNA levels than empty vector and SUD-NM expression in A549 cells (Figure 2D). The results indicated that SUD-MC subdomain significantly involved in SARS-CoV SUD-induced activation of CXCL10 expression in human lung epithelial cells.

### 2.2. SUD-MC Subdomain Activated AP1-Mediated Activation of the CXCL10 Promoter

**Figure 3:**
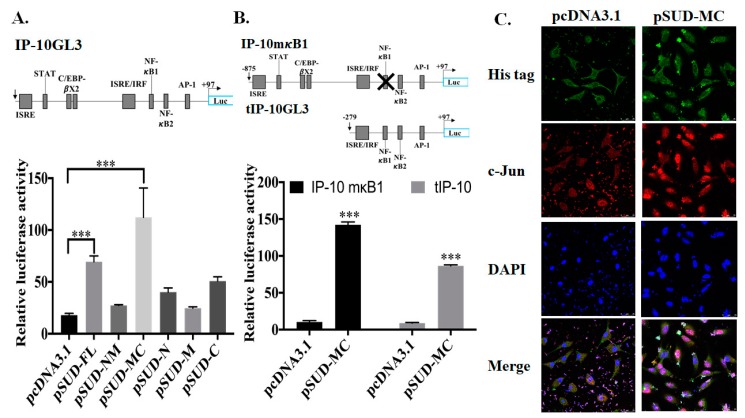
Promoter activation of CXCL10 expression in A549 cells induced by SUD-MC subdomain. Three firefly luciferase reporters including the full length CXCL10 promoter (IP-10GL3) ((**A**), top), NF-κB1 deletion CXCL10 promoter (IP-10mκB1 ((**B**), top), and truncated CXCL10 promoter (tIP-10GL3) ((**B**), top) were used for the dual luciferase reporter assay to determine the CXCL10 promoter activity in transiently transfected cells with pcDNA3.1/His (**C**), pSUD-FL, pSUD-NM, pSUD-MC, pSUD-N, pSUD-M and pSUD-C, respectively. Relative firefly luciferase activity was normalized by internal control renilla luciferase activity 48 h post transfection. In addition, the transiently transfected cells were executed using the immunofluorescent staining with mouse anti-His mAb plus anti-mouse IgG conjugated with FITC (green fluorescence), and rabbit anti-c-Jun mAb plus anti-rabbit IgG conjugated with Alexa 555 (red fluorescence). After the nuclear staining with DAPI (blue fluorescence), the images were photographed by confocal microscopy (Leica TCS SP8X). ***, *p* value < 0.001 compared with pcDNA3.1/His C transfected cell.

Wild type (IP-10GL3), NF-κB site mutant (IP-10mκB1) and truncated (tIP-10GL3) firefly luciferase reporters of CXCL10 promoter were used to examine the transcriptional factor binding region for SUD-mediated activation of CXCL10 promoter (Figure 3A,B). The dual luciferase reporter assays with an internal control Renilla luciferase reporter and wild type CXCL10 promoter driven firefly luciferase reporter indicated that SUD-MC subdomain significantly trigger a greater than 2.2~6.2-fold increases of CXCL10 promoter driven firefly luciferase activity compared to empty vector, NM, N, M, and C subdomains in A549 cells (Figure 3A). In the dual luciferase reporter assays, the activity of mutated and truncated CXCL10 promoter driven firefly luciferase discovered that ISRE/IRF and AP-1 binding sites could be responsible for SUD-MC-induced CXCL10 promoter activation (Figure 3B). To further investigate the nuclear localization of Signal Transducer And Activator Of Transcription 1 (STAT1), STAT2, IFN regulatory factor 1 (IRF1), IRF-3, and c-Jun, transiently transfected cells with pcDNA3.1/His C and pSUD-MC, respectively, were assessed using immunofluorescence staining with indicated primary antibodies, plus DAPI nuclear counterstain (Figure 3C). Imaging analysis of immunofluorescence stained cells indicated that a considerable amount of c-Jun, but STAT1, STAT2, IRF-1, and IRF-3, was translocalized into the nucleus in SUD-MC expressing cells, but a slight amount of c-Jun was in the nucleus of vector transfected cells (Figure 3C). The result displayed that SUD-MC activated AP-1-mediated transcription of CXCL10 gene in A549 cells.

### 2.3. SUD-MC Significantly Induced the Pulmonary Infiltration of Immune Cells and Caused Lung Injury in Mice

**Figure 4:**
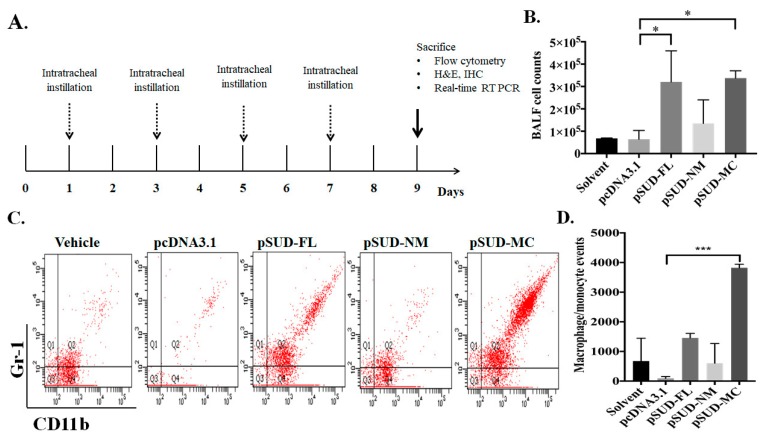
A mouse model using intratracheally instilled with the recombinant plasmids pSUD-FL, pSUD-NM, and pSUD-NM. Wild type C57BL/6 (n = 5/group) were intratracheally instilled with solvent, pcDNA3.1/His C, pSUD-FL, pSUD-NM and pSUD-MC four times every two days (**A**). Total and differential cell counts in bronchoalveolar lavage fluid (BALF) were determined using a hemocytometer at Day 9 (**B**). Moreover, the BALF cells were incubated with APC/Cy7-anti-CD45 antibody-, APC-anti-CD11b, FITC-anti-Gr-1, and PE-anti-F4/80 for 30 min at room temperature in the dark room, and then analyzed by flow cytometry (**C**). The number of macrophages/monocytes (Gr-1^+^/CD11b^+^/F4/80^+^) in BALF was gaged (**D**). *, *p* value < 0.05; ***, *p* value < 0.001 compared to the group instilled with pcDNA3.1/His C.

**Figure 5:**
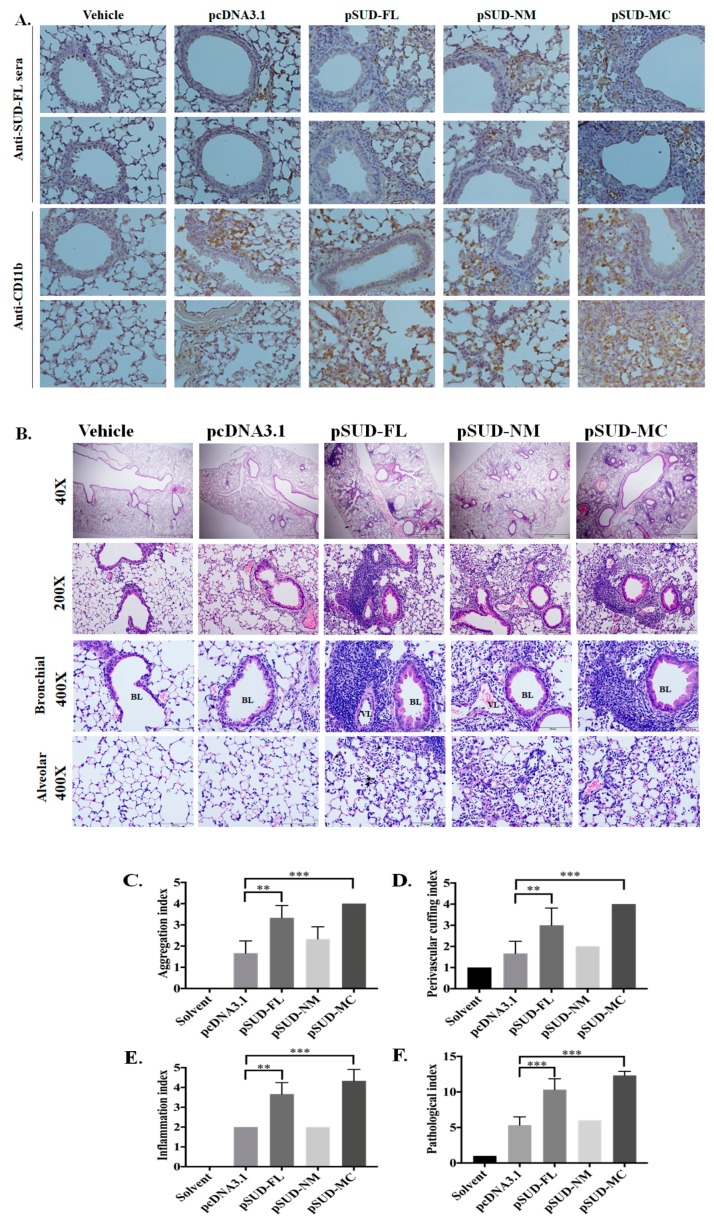
Histopathological changes in the lung tissues of the wild type mice intratracheally instilled with pSUD-FL, pSUD-NM, and pSUD-MC. The sections of the lung tissues from the instilled mice were performed using immunohistochemistry staining with anti-SARS-CoV SUD ((**A**), first and second rows), or anti-mouse CD11b ((**A**), third and fourth rows), and biotinylated universal antibodies from a VECTASTAIN^®^ Elite^®^ ABC Universal Kit (**A**). The sections were also assessed with H&E staining, which the wide range of lung tissues was observed by a light microscope at 40× and 200× magnification ((**B**), first and second rows); inflammatory cells infiltrated around the peribranchial and perivascular, and cell accumulation in alveolar space were examined using a light microscope at 400× magnification ((**B**), third and fourth rows). The indices of histopathological changes in aggregation (**C**), perivascular cuffing (**D**), inflammation (**E**), and pathological index (**F**) were scored based on the degree of lesions ranged from one to five depending on severity: 1 = minimal (<1%); 2 = slight (1–25%); 3 = moderate (26–50%); 4 = moderate/severe (51–75%); 5 = severe/high (76–100%), definite by animal disease diagnostic center (NCHU). ** *p* value < 0.01; ***, *p* value < 0.001 compared to the group instilled with pcDNA3.1/His C. Scale bar, 50 μm.

To examine whether SUD-MC subdomain triggers the infiltration of immune cells into the lung, the mice were intratracheally instilled with the solvent (transfection reagent), pcDNA3.1/His C (vector control), pSUD-FL, pSUD-NM, and pSUD-MC, respectively (Figure 4A). After the instillation (four times, every two days), the mice were sacrificed at 9 days post instillation, and the bronchoalveolar lavage fluids (BALFs) were harvested in order to count and characterize the immune cells in the lung (Figure 4B–D). Total cell counting analysis indicated that total cell counts in the BALFs of mice intratracheally instilled with pSUD-FL and pSUD-MC reached up to 3 × 10^5^ cells/mL, showing a significantly higher increase compared with the other groups with solvent, pcDNA3.1/His C, and pSUD-NM (Figure 4B). In addition, flow cytometry analysis of BALF cells demonstrated that the mice intratracheally instilled with pSUD-MC had a significant increase of the macrophage/monocyte population in the BALFs (Gr-1^+^/CD11b^+^/F4/80^+^) gate (Figure 4C), but not the neutrophil papulation (Gr-1^+^/CD11b^+^/F4/80^−^) gate and the lymphocytes (CD45^+^/CD3^+^), compared to the other groups. Moreover, flow cytometry assay revealed that the count of macrophage/monocyte cells was significantly elevated in the BALFs of the mice instilled with pSUD-MC, but not the other groups (Figure 4D). To confirm the recruitment of macrophage/monocyte infiltration into the mouse lungs, the lung tissues from all groups were subsequently assessed using the immunohistochemistry (IHC) staining (Figure 5A). The assay of IHC staining with immunized sera against His-tagged SUD displayed the protein expression of CoV SUD and its subdomains in the lung tissues from the mice instilled with pSUD-FL, pSUD-NM, and pSUD-MC, respectively (Figure 5A, top two rows). IHC staining with anti-CD11b-specific antibodies showed that SUD-MC triggered a higher infiltration of CD11b positive cells (macrophages/monocytes) in the bronchoalveolar space and accumulated around the pulmonary blood vessels of the instilled mice than full-length SUD, SUD-NM, and pcDNA3.1/His C (Figure 5A, bottom two rows). Therefore, the results specified that SUD-MC subdomain significantly recruited the migration of macrophage/monocyte cells into the bronchial and lung interstitial spaces in the instilled mice.

To further determine the effect of the SUD-MC subdomain on inducing pulmonary inflammation, the histological changes of the mouse lungs from all groups were examined using the H&E staining (Figure 5B). The mice instilled with pSUD-FL and pSUD-MC exhibited severe diffuse alveolar damage symptoms, in which discovered the perivascular (PV) and peribranchial (PB) inflammatory cells infiltration, PV and PB cuffing, edema, and an obvious increase in diameter of alveolar septal injury (Figure 5B). In addition, the SUD-NM subdomain triggered a slight pulmonary inflammation; vector and solvent controls showed no significant alteration in the lung tissues of instilled mice (Figure 5B). Pathology scoring indices of the mouse lung tissues using H&E staining indicated that SUD and its SUD-MC subdomain initiated a significant increase in the indices of cell aggregation, perivascular cuffing, inflammation, and pathogenesis within the mouse lungs compared to the other groups (Figure 5C–F). These results demonstrate that the SUD-MC subdomain substantially caused the severe diffuse alveolar damage symptoms in mice.

### 2.4. SARS-CoV SUD-MC Activated NLRP3 Inflammasome-Mediated Up-Regulation of CXCL10 in Pulmonary Inflammation

**Figure 6:**
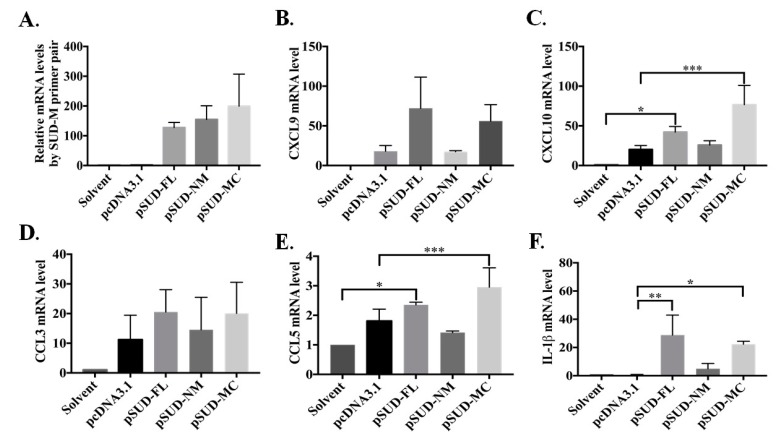
Chemokine expression profile in the lung tissues of the mice in responses to full-length SUD and SUD-MC subdomain. The relative mRNA levels of SUD-M (**A**), CXCL9 (**B**), CXCL10 (**C**), CCL3 (**D**), CCL5 (**E**), and IL-1β (**F**) in lung tissues of the mice intratracheally instilled with indicated plasmids were examined by real-time PCR, normalized by GAPDH mRNA, and presented as the relative ratio. *, *p* value < 0.05; **, *p* value < 0.01; ***, *p* value < 0.001 compared to the group instilled with solvent or pcDNA3.1/His C.

To prove the chemokine expression profile of the lung tissues from the instilled mice, the relative mRNA levels of SUD-M, CXCL9, CXCL10, CCL3, CCL5, and IL-1β in the lung tissues were assessed using real-time PCR assays (Figure 6). The profile of chemokine expression in vivo also indicated that the mice instilled with pSUD-FL and pSUD-MC, but not pSUD-NM and pcDNA3.1/His C, exhibited a significant increase of CXCL10 and CCL5 in the lung tissues (Figure 6C,E). These results reveal that SUD and its SUD-MC subdomain substantially activated the CXCL10 expression in vitro and in vivo. 

**Figure 7:**
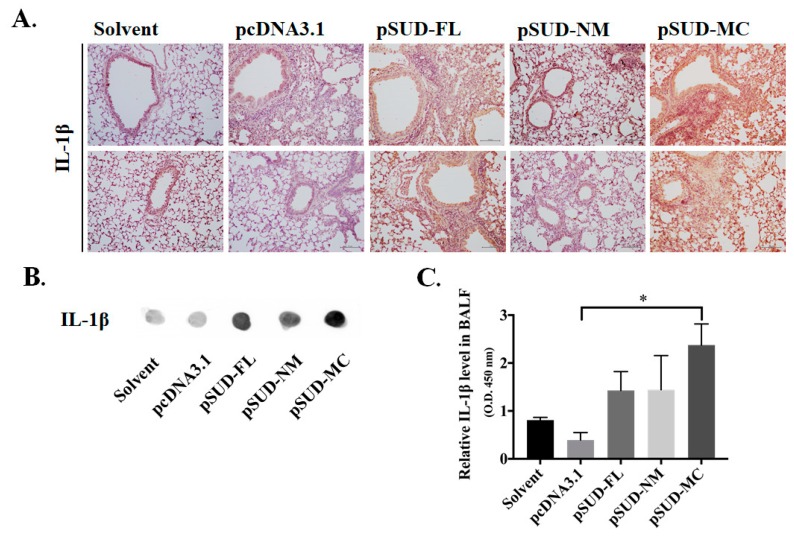
SUD-MC domain induced up-regulation of IL-1β protein levels in the lung tissue and BALF of instilled mice. The protein levels of IL-1β expression in the lung tissues of instilled mice were determined by IHC staining with anti-IL-1β antibody (**A**). Furthermore, semi and relative quantification of the secreted IL-1β in the BALFs were performed using the dot-blot assay with anti-IL-1β (**B**) and direct-ELISA assay (**C**), respectively. *, *p* value < 0.05 compared to the vector control group.

Interestingly, the SUD-MC subdomain also prompted IL-1β expression in the lung tissues of the instilled mice (Figure 6F). Thus, the protein level of IL-1β in lung tissues of instilled-mice were further examined (Figure 7). IHC staining of the lung sections with anti-IL-1β antibody discovered that full-length SUD and its MC subdomain markedly increased the protein level of IL-1β in the mouse lung tissues in comparison with solvent, vector controls and SUD-NM (Figure 7A). Moreover, IL-1β secretion in BALFs was examined using dot blot and direct-ELISA assays with anti-IL-1β antibody (Figure 7B,C). The intensity of dot blots displayed the higher concentration of the secreted IL-1β in BALFs of instilled mice with pSUD-FL and pSUD-MC than other groups (Figure 7B). In the directed ELISA assay, SUD-MC subdomain triggered the significant increase in the IL-1β secretion in BALFs of instilled mice compared with vector control group (Figure 7C). The results showed that SUD-MC also initiated the expression of IL-1β in the lung tissues, as linked with the IL-1β secretion in BALFs responsible for the induction of pulmonary inflammation.

**Figure 8:**
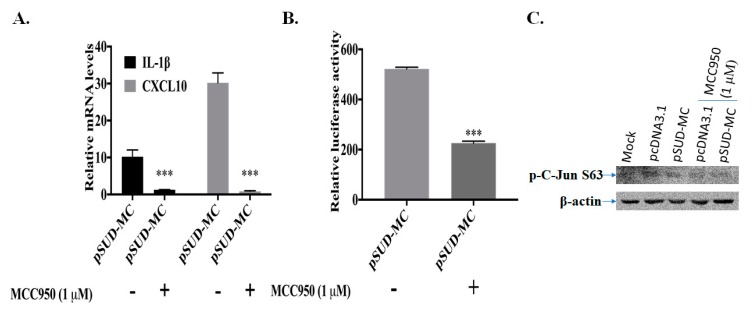
Inhibitory effect of NLRP3 inflammasome inhibitor MCC950 on SUD-MC-induced CXCL10 up-regulation in human lung epithelial cells. The transiently transfected A549 cells with pcDNA3.1/His C or pSUD-MC were treated with or without MCC950 (1 μM) for 48 h, and then harvested for measuring relative mRNA levels of IL-1β and CXCL10 gene by real-time PCR (**A**), detecting the activity of wild type CXCL10 promoter using dual-luciferase reporter assay (**B**) and testing the phosphorylation levels of c-Jun at Serine 63 by Western blotting (**C**). ***, *p* value < 0.001 compared to the transfected cells without the treatment of MCC950.

Since the activation of NLRP3 inflammasome-mediated IL-1β secretion was involved in up-regulating the CXCL10 expression in the alveolar epithelial cells [22], NLRP3-inflammasome inhibitor MCC950 were further used to examine the association between NLRP3 inflammasome activation and CXCL10 up-regulation in human lung epithelial cells (Figure 8). The real-time PCR assays specified the IL-1β upregulation in human lung epithelial cells transfected with pSUD-MC (Figure 8A). The treatment with MCC950 notably reduced the mRNA levels of CXCL10 and IL-1β in SUD-MC-expressing lung epithelial cells (Figure 8A). Meanwhile, dual-luciferase reporter assays demonstrated that MCC950 markedly suppressed SUD-MC-induced transcriptional activity of CXCL10 promoter (Figure 8B). The Western blot assay also discovered that MCC95 significantly reduced SUD-MC-induced phosphorylation of c-Jun at Serine63 (kDa 48) (Figure 8C). The results showed that NLRP3-inflammasome inhibitor MCC950 inhibited c-Jun mediated up-regulation of CXCL10 expression in human lung epithelial cells induced by SARS-CoV SUD-MC.

**Figure 9:**
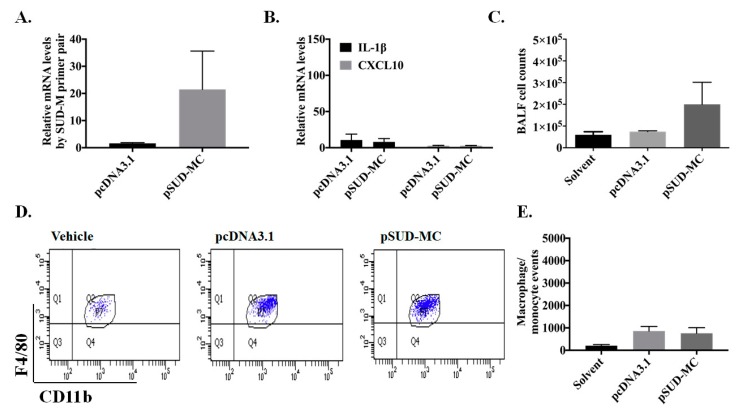

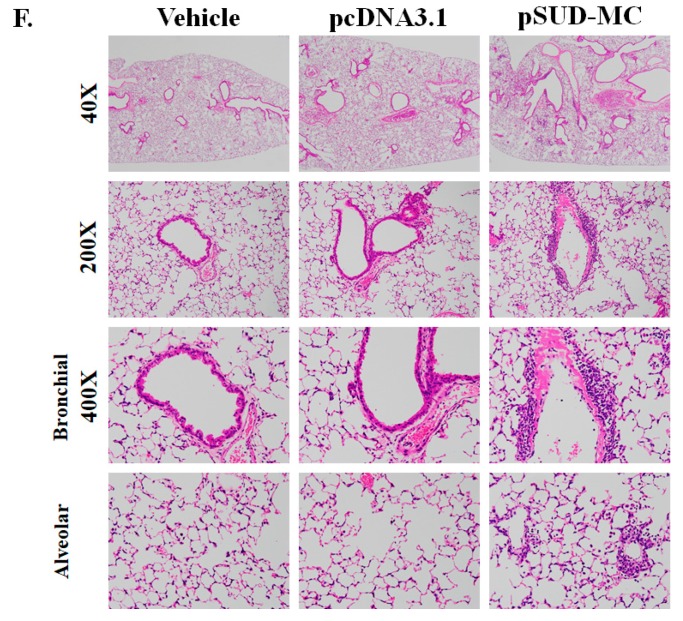
Pulmonary pathology of NLRP3^−/−^ mice in response to SARS CoV SUD-MC. The lung tissues and BALFs of NLRP3^−/−^ mice intratracheally instilled with pSUD-MC or pcDNA3.1/His C were collected for analyzing the relative mRNA levels of SUD-M (**A**), CXCL10, and IL-1β (**B**) using real-time PCR, quantitating the total cell counts in BALFs using a hemocytometer (**C**), and discovering the number of macrophages/monocytes by flow cytometry plus the immunofluorescent staining with APC-anti-CD11b and PE-anti-F4/80 (**D**,**E**). In addition, the histopathological changes in the lung of the NLRP3^−/−^ mice were examined based on the tissue section with the H&E staining (**F**), which the images of lung tissues were photographed by a light microscope at 40×, 200×, and 400× magnification. Scale bar, 50 μm.

Because the activation of NLRP3 inflammasome-mediated IL-1β secretion was related in lipopolysaccharide-induced pulmonary inflammation [23], the NLRP3 gene knockout (NLRP3^−/−^) mice were exploited to verify the effect of NLRP3 inflammasome on SUD-MC-induced CXCL10-mediated pulmonary inflammation (Figure 9). In the NLRP3 gene knockout (NLRP3^−/−^) mice, SUD-MC neither activated the expression of IL-1β and CXCL10 in the mouse lung tissues, nor elicited the migration of immune cells into the lung in the mouse model (Figure 9B–E). Furthermore, H&E staining of the mouse lung tissues discovered that the SUD-MC subdomain induced a slight inflammatory response in the lungs of the instilled NLRP3^−/−^ mice (Figure 9F). These results indicate that SARS-CoV SUD-MC subdomain stimulated NLRP3 inflammasome-mediated activation of CXCL10 expression in vitro and in vivo.

## 3. Discussion

Our study showed that SARS-CoV SUD substantially induced a significant up-regulation of chemokines CXCL10 and CCL5 in human lung epithelial cells and the lung tissues of the mouse model (Figure 1 and Figure 6). Moreover, SARS-CoV SUD stimulated the infiltration of macrophages and monocytes into the lung of the mice instilled with the plasmid (Figure 4), resulting in the CD11b-positive cell aggregation around bronchial and alveolar spaces (Figure 5). Meanwhile, the up-regulated expression of chemokines including CXCL10 was hypothesized to be the main pathogenesis of SARS-CoV in the recruitment of immune cells into human lung tissues [9,24]. Previous studies indicated that SARS-CoV was capable of inducing a significant increase of cytokines and chemokines in various human and animal cells and animal models [25,26,27,28,29,30]. The stimulatory effect of SARS-CoV SUD on the pulmonary infiltration of macrophages/monocytes in the mice was related with the lung injury in SARS-CoV infected mice [28,29]. Among SARS-CoV proteins, nonstructural protein 1 (nsp-1) has been identified as inducing NF-κB-mediated activation of CCL5, CXCL10, and CCL3 expression in human lung epithelial cells [31]. Therefore, the finding in this study was the first report demonstrating the important role of SARS-CoV SUD in inducing CXCL10-mediated pulmonary inflammatory responses in vitro and in vivo. 

SUD-MC subdomain, but not SUD-NM, SUD-N, SUD-M, and SUD-C, serves the functional region of SARS-CoV SUD for activating the CXCL10 and IL-1β expression in human lung epithelial cells and the mouse lung tissues (Figure 2,Figure 3 and Figure 6). Dual luciferase reporter assays with wild type, mutant and truncated promoter regions of CXCL10 gene displayed that the transcriptional factor binding regions for STAT1, STAT2, IRF1, IRF3, and AP1 were responsible for SUD-MC induced activation of CXCL10 expression (Figure 3A,B). Additionally, immunofluorescent staining discovered that c-Jun, a subunit of AP-1 transcriptional factor, was specifically translocated into the nucleus in SUD-MC expressing cells (Figure 3C). Interestingly, c-Jun plays the critical role in activating the IL-1-inducible genes, such CXCL10 and IL-1β, in human fibroblasts [32]. 

**Figure 10:**
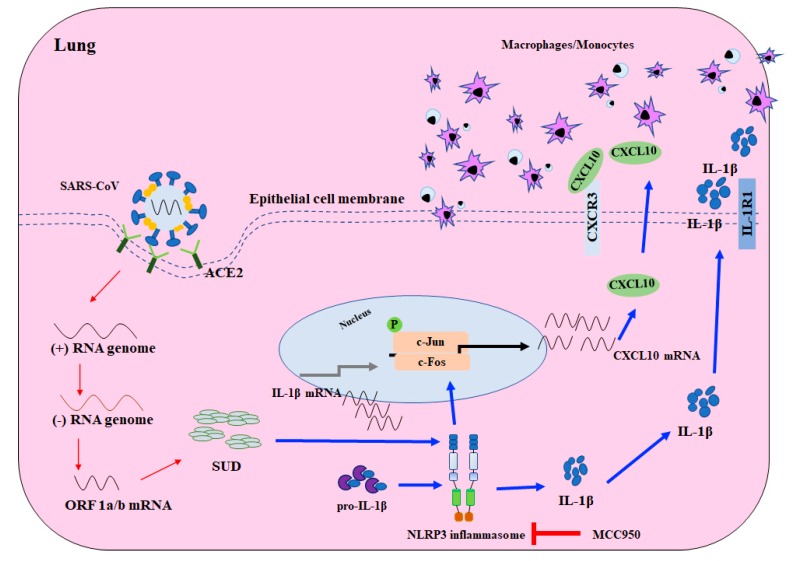
Schematic diagram of SARS-CoV SUD induced CXCL10-mediated pulmonary inflammation through NLRP3 inflammasome pathway. Red arrow; viral transcription and translation, blue arrow; the presumed pathway induced by SARS-CoV SUD.

SUD-MC, a G-quadruplexes binding protein [17], was suggested to exhibit the quadruplex-binding specificity and/or quadruplex-helicase activity that could modulate the internal ribosomal entry site (IRES) initiation for viral translation, the promoter activity for viral transcription, and the mRNA stability of proto-oncogenes, growth factors, transcription, translation factors, etc. [33]. Recently, a synthetic G-quadruplexes oligodeoxynucleotide significantly interfered with NLRP3 Inflammasome-IL1β production in macrophages in response to oxygen-glucose deprivation and lipopolysaccharide stimulation [34]. In this study, SUD-MC significantly initiated the up-regulation of IL-1β expression in the lung tissues, as associated with the secretion of IL-1β in the BALFs of instilled mice (Figure 6 and Figure 7). Moreover, the NLRP3-inflammasome inhibitor MCC950 significantly decreased SUD-MC induced c-Jun mediated activation of IL-1β and CXCL10 expression in human lung epithelial cells (Figure 8). Furthermore, SUD-MC had no significant effect on the induction of IL-1β and CXCL10 expression, the pulmonary infiltration of macrophages/monocytes, and the pathology scoring indices of the lung tissues in the NLRP3^−/−^ mice (Figure 9). Therefore, the results confirmed that SUD-MC stimulated NLRP3 inflammasome-mediated activation of IL-1β and CXCL10 up-regulation in vitro and in vivo, as responsible for the main pathway of SUD-MC-induced pulmonary inflammation. Taken together, our results suggested that NLRP3 inflammasome pathway plays a key role in the activation of AP-1-mediated CXCL10 expression by SARS-CoV SUD-MC, proinflammatory chemokines and cytokines of which, such as CXCL10 and IL-1β, trigger the infiltration of macrophages/monocytes in the lung tissues (Figure 10). Thus, the signal transduction of NLRP3 inflammasome-dependent CXCL10 expression, confirmed by MCC950, might be responsible for SARS-CoV SUD-MC-induced pulmonary inflammation. Importantly, the defect in NLRP3-mediated inflammation in bats is one of the key mechanisms for a special reservoir host of many viruses, including SARS-CoV-1 and 2, MERS-CoV [35]. The finding in NLRP3 inflammasome-mediated activation of IL-1β and CXCL10 up-regulation induced by SARS-CoV SUD might be a valuable target in the development of new anti-SARS agents.

In conclusion, SARS-CoV SUD significantly induced the CXCL10-mediated pulmonary inflammation in vitro and in vivo. SUD-MC subdomain plays the crucial region to activate c-Jun-mediated transcriptional activity in up-regulating the CXCL10 expression in human lung epithelial cells. In the mouse model intratracheally instilled with the recombinant plasmids, SUD-MC pointedly stimulated the infiltration of macrophages/monocytes into the lung, triggered CXCL10-mediated pulmonary inflammation with the severe diffuse alveolar damage symptoms. Moreover, SUD-MC actuated NLRP3 inflammasome-dependent pulmonary inflammation, as confirmed by the assays with NLRP3 inflammasome inhibitor and the NLRP3^−/−^ mouse model. This study demonstrated the biological activity of SARS-CoV SUD in modulating NLRP3 inflammasome-dependent CXCL10-mediated pulmonary inflammation, which provides the potential therapeutic targets for developing the agents against the coronavirus-induced diseases.

## 4. Materials and Methods

### 4.1. Cells 

Human alveolar basal epithelial A549 cells (ATCC CRM-CCL-185) were grown in Dulbecco’s Modified Eagle’s Medium (DMEM; Gibco, Life Technologies, Waltham, MA, USA) supplemented with 10% fetal bovine serum (FBS; Biological Industries, Kibbutz Beit Haemek, Israel), 100 U/mL penicillin and 100 μg/mL streptomycin, and 1μg/mL amphotericin B (HyClone, GE Healthcare Life Sciences, Pittsburgh, PA, USA) in a 5% CO_2_ incubator at 37 °C. A549 cells stably transfected with pSUD-FL, pSUD-NM or SUD-MC were maintained in above media plus 700 μg/mL G418.

### 4.2. Plasmids

The nucleotide sequences of SARS-CoV full-length SUD, SUD-NM, and SUD-MC within the SARS-CoV genome nucleotides 3887–4941, 3887–4673, and 4311–4880 (GenBank accession no. AY291451), respectively, were amplified using PCR with specific primer pairs. SARS-CoV replicon [36] kindly provided by Zhengli Shi (Wuhan Institute of Virology, Chinese Academy of Sciences, Wuhan, China) was used as the template. The primer pairs are shown in Table 1 and used for cloning the full-length SUD, SUD-NM, SUD-MC, SUD-N, SUD-M and SUD-C subdomains, respectively. The PCR products with a restriction site *KpnI* at 5′ end and a restriction site *Xho*I at 3′ end were cloned into the expression vector pcDNA3.1/His C (Invitrogen, Carlsbad, CA, USA) after double digest reaction with *KpnI* and *Xho*I, and then the resultant recombinant plasmids were named as pSUD-FL, pSUD-NM, pSUD-MC, pSUD-N, pSUD-M, and pSUD-C, respectively. In addition, firefly luciferase reporter plasmids IP-10GL3, tIP-10GL3, and IP-10mκB1, as gifted from David Proud at University of Calgary (Canada), contain wild type (−875/+97), truncated (−279/+97), and NF-κB1 site mutant CXCL10 promoter, respectively.

### 4.3. Transient Transfection and Stable Clone Cell Line Generation 

A549 cells (2 × 10^5^) were seeded in 6-well plate overnight, and transfected with 2.5 μg of pcDNA3.1/His C, pSUD-FL, pSUD-NM, or pSUD-MC in 6 μL jetPRIME reagent (Polyplus-transfection S.A, Illkirch, France). After 24-h or 48-h incubation, the transfected cells were harvested for the further assays. For generating the cell lines, the transfected cells were selected in above media containing 700 μg/mL G418 (Sigma-Aldrich, Saint Louis, MO, USA) for 1 month. Stable cell lines expressing SUD-FL, SUD-NM, and SUD-MC were analyzed using qRT-PCR and immunofluorescence staining.

### 4.4. Real-Time RT-PCR 

The total RNAs were purified from transfected cells, stable cell lines and mouse lung tissues using the PureLink Micro-to-Midi Total RNA Purification System kit (Invitrogen, Carlsbad, CA, USA). Relative mRNA levels of host chemokines and viral SUD were analyzed using a SuperScript™ III Platinum^®^ Two-Step qRT-PCR Kit with SYBR^®^ Green (Invitrogen, Carlsbad, CA, USA). After reverse transcription by SuperScript III RT, the real-time PCR was performed with the cDNAs, specific primer pairs, and Platinum^®^ SYBR^®^ Green qPCR SuperMix according to the amplification protocol consisting of 1 cycle at 50 °C for 2 min, 1 cycle at 95 °C for 10 min, 45 cycles at 95 °C for 15 sec, and 60 °C for 1 min. Primer pairs for the detection mRNA levels of human and mouse chemokines (CXCL8, CXCL10, CXCR3, CCL-2/MCP-1, CCL3/MIP-1α, and CCL5/RANTES) as well as SARS-CoV SUD, SUD-NM, and SUD-MC are listed in Table 2. Relative mRNA levels of indicated genes were standardized by human β-actin and mouse GAPDH and then calculated by the comparative CT method (ΔΔCT method).

### 4.5. Dual-Luciferase Reporter Assay

To examine the activation of IP-10 promoter by SARS-CoV SUD, mock and transfected cells expressing SUD-FL, SUD-NM, and SUD-MC were co-transfected with CXCL10 promoter-driven firefly luciferase reporter plasmids (IP-10GL3, tIP-10GL3, and IP-10mκB1) and an internal control Renilla luciferase reporter pRluc-C1, as described in our prior report [37]. After a 1-day incubation, the cells were harvested and then dissolved in the lysis buffer; the activity of firefly and Renilla luciferases in the lysate was detected using Dual-Luciferase^®^ Reporter (DLR™) Assay System (Promega, Waltham, MA, USA) in Clarity™ Luminescence Microplate Reader (BioTek Instruments, Winooski, VT, USA).

### 4.6. Immunofluorescence Staining

To examine the SUD expression, stable cell lines transfected with pcDNA3.1/His C, pSUD-FL, pSUD-NM, and pSUD-MC, respectively, were seeded on the sterilized coverslip placed in the well of a 24-well plate. After 2-day incubation or treatment with the kinase inhibitors, the cells were fixed by 3.7% paraformaldehyde in PBS for 1 h, permeabilized by 0.1% Triton X-100 in PBS for 10 min, and then quenching by 15 mM NH4Cl. Blocking with 1–2% bovine serum albumin (BSA) buffer for 1 h at room temperature. Subsequently, the cells were incubated with anti-*E. coli* synthesized SUD mouse polyclonal antibody, mouse anti histidine tag antibody (MCA1396; Bio-Rad, Hercules, CA, USA), anti-c-Jun (ab32137; Abcam), anti-IRF-1 (ab186384; Abcam, Cambridge, MA, USA) and anti-NF-κB p65 (ab32536; Abcam) mouse monoclonal antibodies at 4 °C overnight, reacted with secondary antibodies conjugated with Alexa Fluor^®^ 488 (ab150077; Abcam) or goat Anti-mouse IgG H&L FITC (ab6785; Abcam) or goat anti-Rabbit IgG (H+L) Alexa Fluor 555 (A-21428; Invitrogen, ThermoFisher Scientific, Waltham, MA, USA) in dark for 1h at room template. After washing cells for three times, the cells on the coverslip were mounted using the Fluoromount-G mounting medium with DAPI (ThermoFisher Scientific) under slides, and then photographed by Olympus, BX50 immunofluorescence microscopy (Olympus, Tokyo, Japan) and Leica TCS SP8 X Confocal Spectral Microscope Imaging System with White Light Laser.

### 4.7. Direct Enzyme-Linked Immunosorbent Assay 

100 μL BALF of each mouse was quadruple added into the 96-well plates, mixed with 100 μL coating buffer, and then incubated at 4 °C overnight. After washing three times with 1x TBST, the pre-coating plate was subsequently blocked with 5% fat-free in 1xTBST at room temperature for 1 h, washed with 1xTBST, incubated with rabbit anti-IL-1β mAb (ab9722; Abcam) for 1 h, and reacted with HRP-conjugated goat anti-rabbit IgG antibody. Finally, the immune-reactive complexes were reacted with KPL SureBlue™ TMB Microwell Peroxidase Substrate (SeraCare, West Bridgewater, MA, USA) plus the stop buffer (1N HCl), and recorded by measuring the optical absorbance at 450 nm using the ELISA plate reader SpectraMax iD3 (MOLECULAR DEVICES, Wals, Austria).

### 4.8. Western Blot and Dot Blot Assays

To determine the phosphorylation of c-Jun in at Serine63 in response to SUD-MC, transiently transfected A549 cells with empty vector pcDNA3.1/His C or pSUD-MC were harvested 48 h post transfection. The cell lysate was mixed with SDS-PAGE sample buffer for boiling 10 min, and then loaded in 10% SDS-PAGE [37]. After running the gel electrophoresis, the proteins were electrophoretically transferred onto the nitrocellulose membrane that was further blocked using the blocking solution (5% fat-free milk) overnight and reacted with primary antibodies anti-Phospho-c-Jun (Ser63) mAb (#2361; Cell Signaling), anti-IL-1β mAb (ab9722; Abcam), and anti-β-actin mAb (NB600-501; Novus). The immune-reactive bands were probed with HRP-conjugated goat anti-mouse or rabbit IgG antibodies, and then detected by enhanced chemiluminescent HRP substrate (Amersham Pharmacia Biotech, Little Chalfont, United Kingdom). For dot blot assay, 15 μL of BALF was spotted onto the NC membrane for air drying for 15 min, and then evaluated by following the procedure of Western blot assay.

### 4.9. Animal Model

The protocol of the mouse model was reviewed and approved by the Institutional Animal Care and Use Committee (IACUC) at China Medical University on December 24th, 2015 (Animal Use Protocol No. CMUIACUC-2016-049-2). Five 6-week-old female wild type or NLRP3 knockout (NLRP3^−/−^) C57BL/6 mice were anesthetized via giving isoflurane under 2-3 cc/min air^*100^ flow rate by the Matrx VMS^®^ small animal anesthesia machine (Midmark, Tampa, FL, USA) for 5 to 10 minutes. 25 μg of the plasmid (pcDNA3.1/His C, pSUD-FL, pSUD-NM or pSUD-MC) in 50 μl of the in vivo-jetPEI^®^ agent (Polyplus-transfection S.A, Illkirch, France) was loaded in a micro syringe (Hamilton^®^, Salt Lake City, UT, USA), and intratracheally instilled into the mouse lungs. After 4 intratracheal instillations with the plasmid every 2 days, the mice were sacrificed for harvesting the bronchoalveolar lavage fluid (BALF) for total cell counting and flow cytometry assays, and collecting the lungs, which left lobe was performed by Haemotoxylin and Eosin (H&E) and immunohistochemistry (IHC) staining assays and right lobes were homogenized for qRT-PCR assay.

### 4.10. Flow Cytometry Assay

The immune cells in the BALFs of each mouse was directly counted using a hemocytometer, and then collected after the centrifugation at 716 (×g) for 5 min. The cells were resuspended and reacted with primary antibodies, including APC/Cy7 anti-mouse CD45 (biolegend, San Diego, CA), APC anti-human/mouse CD11b (biolegend, San Diego, CA), FITC anti-mouse Ly 6G/Ly 6C (Gr-1) (biolegend, San Diego, CA), PE anti-mouse F4/80 (biolegend, San Diego, CA), PE anti-mouse CD3ε (biolegend, San Diego, CA), FITC anti-mouse CD4 (bio legend, San Diego, CA) and APC anti-mouse CD8a (biolegend, San Diego, CA). After a 15-min incubation, the cells were washed with PBS twice, fixed with 2% paraformaldehyde, and then detected using flow cytometry under a BD FACSCanto system (Becton Dickinson FACS Calibur, Franklin Lakes, NJ, USA). 100,000 live-cell events per sample were analyzed by BD FACS Canto Clinical and FACS Diva software (Becton Dickinson, Franklin Lakes, NJ, USA), in which the Gr-1^+^/CD11b^+^/F4/80^+^ cells gated in Q2 were macrophages/monocytes, the Gr-1^+^/CD11b^+^/F4/80^−^ cells gated Q2-1 from CD45^+^ cells were neutrophils, and the CD45^+^/CD3^+^ cells gated from P1 were lymphocytes.

### 4.11. Histopathology and Immunohistochemistry Assays

The mouse lung tissues were fixed in 10% neutral buffered formalin overnight, dehydrated in 70% ethanol for 30 min, in 95% ethanol for 30 min, and in 100% ethanol for 30 min, and then embedded in paraffin at 58 °C. The paraffin embedded sections at the thickness of 4-15 µm were obtained by the rotary microtome, deparaffinized with xylene 3 times for 5 min, rehydrated from 100% ethanol to 80% ethanol, washed with distilled water for 5 min, and then stained with Hematoxylin Gill II (Leica Surgipath, Buffalo Grove, IL, USA) for 8 min and counterstained with Eosin (Leica Surgipath) for 1 min. Finally, the stained sections were dehydrated with 100% ethanol, cleared in xylene three times for 5 min, dried at room temperature in air, and mounted by Micromount (Leica Surgipath). In the IHC staining assay, the sections were deparaffinized, rehydrated, and then heated in the Tris-EDTA Buffer (pH 9.0) at 100 °C for 20 min. Subsequently, the sections were blocked with the block solution (1–3% protein) for 2 h, incubated with primary antibodies (anti-SARS-CoV SUD and anti-mouse CD11b (ab133357; Abcam)) at 4 °C overnight, treated in 3% H_2_O_2_ for 10 min to quench the endogenous peroxidase activity, and reacted with biotinylated universal antibodies from a VECTASTAIN^®^ Elite^®^ ABC Universal Kit (Vector Laboratories, Burlingame, CA, USA) for 30 min. After incubating with VECTASTAIN elite ABC reagent 30 min, the dark brown-black precipitate of the immune complexes in the sections was developed using DAB Peroxidase Substrate Kit (Vector Laboratories). Finally, the sections were counterstained with hematoxylin for 2-3 min, and then mounted by Micromount (Leica).

### 4.12. Inhibitor Treatment

For inhibitor treatment, vector control and SUD-MC-expressing cells were incubated with MCC950 (NLRP3 inflammasome inhibitor) (17510; Cayman Chemical, Ann Arbor, MI, USA) 1 μM, and then harvested for dual-luciferase reporter, real-time RT-PCR, and Western blotting.

### 4.13. Statistical Analysis

All data were calculated from three independent experiments. One-way ANOVA followed by Scheffe’s post-hoc test was used to analyze all data. Statistical significance was considered at the *p* value of less than 0.05.

## Figures and Tables

**Table 1:** Primers used for cloning full-length SUD and its subdomains into the expression vector pcDNA3.1/His C.

Gene Fragments	Primer Name	Primer Sequence
full-length SUD	F-SUD-N	5′-AGTG GGTACC T ATTAAGGCCTGCATTGATGAG-3′
	R-SUD-C	5′-AGTG CTCGAG CG TGTGTGGAGATTAGTGTTGTC-3′
SUD-NM	F-SUD-N	5′-AGTG GGTACC T ATTAAGGCCTGCATTGATGAG-3′
	R-SUD-M	5′-AGTG CTCGAG CG TGACGAAGTGAGGTATCC-3′
SUD-MC	F-SUD-M	5′-AGTG GGTACC T TGGAATTTGAGAGAAATG-3′
	R-SUD-C	5′-AGTG CTCGAG CG TGTGTGGAGATTAGTGTTGTC-3′
SUD-N	F-SUD-N	5′-AGTG GGTACC T ATTAAGGCCTGCATTGATGAG-3′
	R-SUD-N	5′-AGTG CTCGAG CG TAGAATCTCTTCCTTAGC-3′
SUD-M	F-SUD-M	5′-AGTG GGTACC T TGGAATTTGAGAGAAATG-3′
	R-SUD-M	5′-AGTG CTCGAG CG TGACGAAGTGAGGTATCC-3′
SUD-C	F-SUD-C	5′-AGTG GGTACC T TCAAAGACATCTGAGGAG-3′
	R-SUD-C	5′-AGTG CTCGAG CG TGTGTGGAGATTAGTGTTGTC-3′

**Table 2:** Primer list for real-time PCR.

Species	Gene	Forward Primer Sequences	Reverse Primer Sequences
SARS-CoV	SUD-N	TCAGAACATGCTTAGAGG	TGGAGGGTATTACAACACAA
	SUD-M	CATGCTGAAGAGACAAGAAAAT	AGTATAAAAGAAGAATCGGACACC
Human	IL-1β	ATCACTGAACTGCACGCTCC	TTGTTCTCCATATCCTGTCCC
	CXCL8	CGATGTCAGTGGATAAAGACA	TGAATTCTCAGCCCTCTTCAAAAA
	CXCL9	CGTGGTAAAACACTTGCGGATATT	CAATCATGCTTCCACTAACCGACT
	CXCL10	CCAATTTTGTCCACGTGTTG	TTCTTGATGGCCTTCGATTC
	CCL3	AGCTGACTACTTTGAGACGAGCA	CGGCTTCGCTTGGTTAGGA
	CCL5	TCCCCATATTCCTCGGAC	GTCTAGAGGAACCGGTGTTAC
	β-actin	AGGCCACCCCAGAGGACAAC	CCAGAGGCGTACAGGGATA
Mouse	IL-1β	CCAGCAGGTTATCATCATCATCC	CTCGCAGCAGCACATCAAC
	CXCL9	GCCATGAAGTCCGCTGTTCT	GGGTTCCTCGAACTCCACACT
	CXCL10	GACGGTCCGCTGCAACTG	GCTTCCCTATGGCCCTCATT
	CCL3	TGAAACCAGCAGCCTTTGCTC	AGGCATTCAGTTCCAGGTCAGTG
	CCL5	GATGGACATAGAGGACACAACT	TGGGACGGCAGATCTGAGGG
	GAPDH	TGAGGCCGGTGCTGAGTATGTCG	CCACAGTCTTCTGGGTGGCAGTG
